# Thanks

**Published:** 1888-11

**Authors:** 


THANKS.—THE BUFFALO MEDICAL AND SURGICAL JOURNAL commenced a new volume with the August number in a new and much improved dress.—*St. Louis Courier of Medicine, October.*

The University Medical Magazine, published in the interests of the University of Pennsylvania, made its appearance last month The second number—November—has come to hand promptly, as might be expected from the able editorial staff, advisory and active, which stands at the helm. It is a creditable addition to the periodical medical literature of the country.

